# Identification of coordinately regulated microRNA-gene networks that differ in baboons discordant for LDL-cholesterol

**DOI:** 10.1371/journal.pone.0213494

**Published:** 2019-03-15

**Authors:** Genesio M. Karere, Jeremy P. Glenn, Shifra Birnbaum, Roy Garcia, John L. VandeBerg, Laura A. Cox

**Affiliations:** 1 Department of Genetics, Texas Biomedical Research Institute, San Antonio, TX, United States of America; 2 Department of Internal Medicine, Wake Forest School of Medicine, Winston-Salem, NC, United States of America; 3 Department of Human Genetics and South Texas Diabetes and Obesity Institute, School of Medicine,The University of Texas Rio Grande Valley, Brownsville/Harlingen/Edinburg, TX, United States of America; 4 Southwest National Primate Research Center, Texas Biomedical Research Institute, San Antonio, TX, United States of America; Texas A&M University, UNITED STATES

## Abstract

**Rationale:**

Plasma low-density lipoprotein cholesterol (plasma LDL-C), vascular endothelial cells and peripheral blood mononuclear cells (PBMCs), particularly monocytes, play key roles in initiating atherosclerosis, the primary cause of cardiovascular disease (CVD). Although the mechanisms underlying development of atherosclerosis are not well understood, LDL-C is known to influence expression of endothelial microRNAs (miRNAs) and gene-targets of miRNAs to promote cell senescence. However, the impact of LDL-C on expression of PBMC miRNAs and miRNA targeted genes in response to an atherogenic diet is not known. In this study, we used unbiased methods to identify coordinately responsive PBMC miRNA- gene networks that differ between low and high LDL-C baboons when fed a high-cholesterol, high-fat (HCHF) diet.

**Methods and results:**

Using RNA Seq, we quantified PBMC mRNAs and miRNAs from half-sib baboons discordant for LDL-C plasma concentrations (low LDL-C, n = 3; high LDL-C, n = 3) before and after a 7-week HCHF diet challenge. For low LDL-C baboons, 626 genes exhibited significant change in expression (255 down-regulated, 371 up-regulated) in response to the HCHF diet, and for high LDL-C baboons 379 genes exhibited significant change in expression (162 down-regulated, 217 up-regulated) in response to the HCHF diet. We identified 494 miRNAs identical to human miRNAs and 47 novel miRNAs. Fifty miRNAs were differentially expressed in low LDL-C baboons (21 up- and 29 down-regulated) and 20 in high LDL-C baboons (11 up- and 9 down-regulated) in response to the HCHF diet. Among the differentially expressed miRNAs were miR-221/222 and miR-34a-3p, which were down-regulated, and miR-148a/b-5p, which was up-regulated. In addition, gene-targets of these miRNAs, *VEGFA*, *MAML3*, *SPARC*, and *DMGDH*, were inversely expressed and are central hub genes in networks and signaling pathways that differ between low and high LDL-C baboon HCHF diet response.

**Conclusions:**

We have identified coordinately regulated HCHF diet-responsive PBMC miRNA-gene networks that differ between baboons discordant for LDL-C concentrations. Our findings provide potential insights into molecular mechanisms underlying initiation of atherosclerosis where LDL-C concentrations influence expression of specific miRNAs, which in turn regulate expression of genes that play roles in initiation of lesions.

## Introduction

Cardiovascular disease (CVD), the leading cause of morbidity and mortality in developed countries [1,2], is commonly associated with development of atherosclerosis. The pathophysiological characteristics of atherosclerosis are a consequence of interactions between genetic and environmental factors including diet. Clinical and epidemiological studies indicate that atherosclerotic burden is positively correlated with plasma LDL-C concentrations [3–6], and high LDL-C concentration is a major risk factor or atherosclerosis [7].

High LDL-C concentrations contribute greatly to the initiation of atherosclerosis, whereby LDL particles and associated cholesterol activate adhesion molecules on vascular endothelial cells (EC), reduce synthesis of EC-derived relaxing factor, and promote EC apoptosis [7, 8]. It is posited that upon activation of adhesion molecules, PBMCs, particularly monocytes, adhere to the endothelium, infiltrate the neointima through the endothelial layer, phagocytize LDL-C and transform to macrophages, triggering inflammation-signaling cascades. Previous *in vitro* studies demonstrated that LDL-C, particularly oxidized LDL-C (ox-LDL-C), induces EC apoptosis by regulating the expression of genes and miRNAs. For example, ox-LDL-C induces EC apoptosis by down-regulating *BCL2* [9, 10] and the miR-222/221 family [11], while up-regulating miR-365 [12].

Previously we showed that hepatic miR-222/221 was down-regulated in response to feeding a HCHF diet to baboons with high LDL-C concentrations [13]. In the current study, we hypothesized that PBMC miRNAs and miRNA gene-targets are differentially expressed in response to changes in LDL-C concentrations, and that miRNA-gene networks are concomitantly modulated. To test this hypothesis, half-sib adult baboons (*Papio hamadryas*) exhibiting discordant LDL-C plasma concentrations (low and high LDL-C) were challenged for 7 weeks with a HCHF diet. Blood samples were collected before and after the diet challenge. We quantified all PBMC-expressed genes and miRNAs by unbiased RNA Seq.

We identified differentially expressed genes and miRNAs, inversely expressed miRNA gene-targets and their integrated networks, and gene ontologies and biological pathways modulated in response to changes in LDL-C concentrations. By analyzing half-sib baboons discordant for the LDL-C plasma concentrations (> 2 S.D. difference), we minimized genetic variation due to genetic background and increased the likelihood that we would discover miRNAs and miRNA gene-targets that are LDL-C responsive.

The baboon is a well-characterized model for dyslipidemia and atherosclerosis [6, 14] and is very similar to humans both physiologically and genetically [15], suggesting that findings in baboons will translate to humans. In addition to genetic background, we are able to control diet and environment in baboons, which is very difficult in humans. To our knowledge, the influence of LDL-C concentrations on PBMC miRNA and miRNA gene-target expression has not been investigated in primates. Identification of baboon PBMC miRNAs and miRNA gene-targets that are differentially expressed in response to changes in LDL-C concentrations may provide insights into understanding mechanisms by which early atherosclerosis is initiated, potentiating discovery of novel therapeutic targets and biomarkers for dyslipidemia.

## Materials and methods

### Animals

Baboons were bred and maintained at the Southwest National Primate Research Center (SNPRC), Texas Biomedical Research Institute (Texas Biomed), which is accredited by the Association for Assessment and Accreditation of Laboratory Animal Care International. The animals were maintained in outdoor group housing, which provided full social environment and physical activity, and food and drink *ad libitum*. The Texas Biomedical Research Institute’s Institutional Animal Care and Use Committee approved experimental protocols, and SNPRC veterinarians and veterinary staff conducted all procedures, and all efforts were made to minimize suffering. The animals were not euthanized after the experiments.

### Selection of baboon half-sib-pairs discordant for LDL-C

Based on phenotypic and genotypic analysis of 951 pedigreed baboons, we identified three half-sib-pairs with contrasting phenotypes for LDL-C concentrations as described. The members of each half-sib-pair differed by at least two standard deviations for LDL-C concentrations [16].

### Diet challenge

Half-sib baboons were maintained on a chow diet that is low in fat (4% of calories) and cholesterol (0.03 mg/kcal) and challenged for 7 weeks with HCHF diet, which is high in fat (40% of calories) and cholesterol (1.7 mg/kcal). The dietary components and the dietary challenge protocols have been described [17].

### Sample collection

Prior to sample collection, baboons were sedated with ketamine (10 mg/kg), given atropine (0.025 mg/kg) and intubated. Blood pressure was measured by automated arm cuff (Collin), and oxygen saturation, heart and respiration rates were monitored by pulse oximetry. Blood samples (10 ml) were collected by venipuncture in EDTA tubes, before (n = 6) and after the HCHF challenge diet (n = 6). Whole blood was centrifuged at 10,000g for 10 min, and buffy coats were aspirated and stored at -80°C.

### RNA isolation

Total RNA was isolated and purified from buffy coats of 12 samples using TRIzol Reagent and miRNeasy Mini Kit (Qiagen) as described previously [18]. RNA quantity was assessed spectrophotometrically using a NanoDrop 8000 (Thermo Fisher Scientific, Wilmington, DE), and quality was assessed using an Agilent Bioanalyzer 2100 (Agilent, Santa Clara, CA). RNA integrity was assessed by electrophoresis in a denaturing 1% agarose gel and stored at −80°C until further use.

### RNA sequencing and data analysis

Complementary DNA (cDNA) was generated for RNA Seq using Illumina’s mRNA Seq Sample Preparation Kit according to the manufacturer’s protocol and as described previously [18]. Briefly, we enriched for mRNAs using poly-A selection and fragmented the mRNA by chemical fragmentation. We generated the first strand of cDNA using reverse transcription and Illumina’s random hexamer primers, and the second strand using DNA Polymerase I and RNase H. We multiplexed all samples in one pool and hybridized the pool to an individual lane of a flow cell for cluster generation using the Illumina Paired End Cluster Generation Kitv4 (2x100bp) and Cluster Station. Sequencing was performed using the Illumina v4 Sequencing Kit and HiSeq 2500 Sequencer.

The sequencing output FASTQ files (GEO accession number: GSE114722) were analyzed using default settings of Partek Flow (Partek Inc., MO). Briefly, paired sequence reads for each sample were trimmed bases from the 5’ end, followed by trimming of bases from both ends for sequence reads with a Phred score of less than 30.The sequence reads were aligned with STAR aligner v2.3.1j to baboon transcriptome (Ensembl PapAnu2 release 76). To quantify the expression levels of transcripts, the mapped reads were normalized using transcripts per kilobase million (TPM), whereby read counts were divided by the length of a gene in kilobase to compute reads per kilobase (RPK). The sum of all RPK values in a sample were divided by 10^6^ to compute ‘per million scaling factor’. Finally, each RPK was divided by the ‘per million scaling factor’ to obtain TPM. The reads were quantified using Partek’ expectation maximization (EM) algorithm. Differential expression of genes was evaluated using Partek’s gene specific analysis (GSA). The goal of GSA is to identify a statistical model that is the best for a specific gene, and then to use the best model to test for differential expression.

Using GSA, p values and fold-change for each gene, calculated as normalized ratio of mapped reads before and after diet challenge, were outputted. Finally, using Partek Genomic Suite we generated unsupervised hierarchical clustering of profiles of differentially expressed genes.

### Functional enrichment analysis

To enhance the understanding of the biological relevance of all differentially expressed genes before and after HCHF diet challenge in low and high LDL-C baboons, we performed functional enrichment analysis using ClueGO, a plug-in app for Cytoscape [19]. ClueGo facilitates generation of functionally grouped gene ontology and pathway networks and charts to decipher the biological relevance of expressed genes. The statistical test used for the enrichment was based on the both-sided hypergeometric option with a Benjamini-Hoschberg correction and kappa score of 0.4. In addition, we invoked the GO Term Fusion option as well as biological, functional, molecular and immunity GO terms.

To further understand the biological relevance of the genes that were common and inversely expressed in both low and high LDL baboons, we performed network analysis using ConsensusPathDB-human (http://cpdb.molgen.mpg.de/), a web-based analysis tool. The significance of GO terms and pathways was tested with p value set at <0.01, a minimum overlap with input genes set at 2 and a GO level 5 category option.

### Small RNA sequencing and data analysis

We used protocols and procedures described previously [13] and used the same RNA samples that were used for RNA Seq above. After isolating RNA, we enriched the sample with sncRNAs by using mirVana miRNA Isolation Kit (Ambion). We used 2μg of sncRNAs to synthesis cDNA. After purification, the ibraries were quantified using QuBit fluorometry and Nanodrop 8000 spectrometry. Library clones were generated using cBot, and sequenced using Illumina’s Genome Analyzer (GAIIx). Sequence reads analysis and identification of differentially expressed miRNAs were done using mirTools [20]. For the miRNAs, sequence reads (SRA accession number: SRP148696) were normalized using reads per million mapped to miRNA (RPMM), whereby the sum of miRNA specific read counts per sample was divided by 10^6^ to compute ‘per million scaling factor’. Finally, read counts were divided by the ‘per million scaling factor’ to compute RPMM.

### Identification of gene-miRNA integrated networks

To identify miRNA-gene integrated networks, we used MiRNA Target Filter in IPA. Briefly, we uploaded both differentially expressed miRNAs and differentially expressed genes and identified miRNA targets using TargetScanHuman v6.2/Tar-Base/MiRecords software embedded in IPA. TargetScanHuman considers matches to human 3’ UTRs and their orthologs. We used the Build function to connect inversely expressed miRNA-mRNA molecules and generate networks. The low LDL-C network was overlaid on a miRNA-gene list for high LDL baboons to generate a high LDL-C network.

### Statistical analysis

Statistical analysis was performed using algorithms embedded in miRTool for miRNA expression profiles and Partek Genomic Suite for gene expression profiles. Arithmetic means derived from three samples for baboons fed chow or HCHF diet per baboon phenotype were used to identify differentially expressed miRNAs and genes, and analyzed by a two-tailed paired t- test. One sample two-tailed t-test was used to test the mean difference in LDL-C concentrations of the animals before and after diet challenge. Statistical significant threshold was set at p < 0.05. Where appropriate multiple testing analysis was performed to correct p values using Benjamini-Hochberg method described by McDonald JH. (2014) [21].

For the miRNA analysis, we used 5% False Discovery Rate (FDR) and 20% for the gene analysis; meaning that we accepted the possibility that 20% of the genes with p values less than 0.05 are likely false positive. Statistically significant pathways and GO terms were determined using algorithms embedded in IPA, ClueGo, or ConsensusPathDP-human software. P-values <0.05 were considered statistically significant.

## Results

### LDL-C concentrations in low and high LDL-C baboon baboons

Table 1 illustrates changes of LDL-C concentrations after the HCHF diet challenge. For both low and high LDL-C baboons, LDL-C concentrations increased significantly after diet challenge (low, p = 0.01; high, p = 0.03). For the high LDL-C baboons, LDL-C concentrations increased 2-fold compared to low LDL-C baboons after the HCHF diet challenge (p = 0.02).

**Table 1:** Plasma LDL-CLDL-C concentrations before and after HCHF diet challenge in low and high.

LDL-C baboons		
Diet	Chow	HCHF
Low LDL-C Baboons	19.3 (8.1)	31.3 (2.5)
High LDL-C Baboons	43.7 (15.0)	114.3 (19.5)

Standard deviations are indicated in parenthesis.

### Differential expression of PBMC genes responsive to HCHF diet

**Fig 1:**
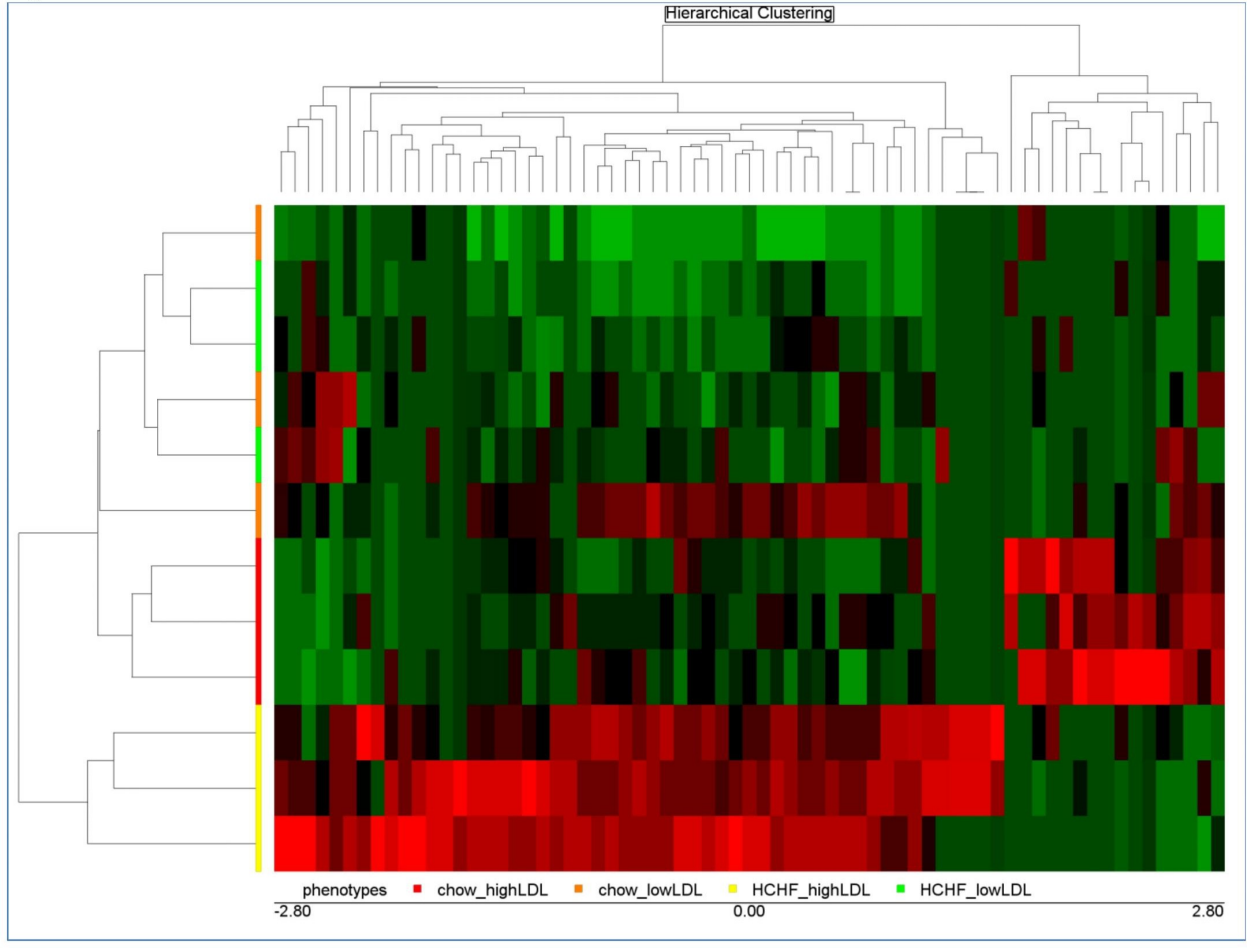
Dendrogram showing unsupervised hierarchical clustering of baboon PBMC gene expression profiles in low and high LDL-C baboons in response to HCHF diet. Columns and rows, respectively, denote genes and time points.

We identified genes that were differentially expressed in response to LDL-C concentration changes by analyzing the expression levels before and after HCHF diet challenge. For low LDL-C baboons, 626 genes (255 down-regulated, 371 up-regulated) exhibited significant change in expression, while 272 genes (114 down-regulated, 158 up-regulated) remained significantly differentially expressed in high LDL-C baboons after p-value correction (S1 Table). Tables 2 and 3 show the top 35 genes with highest change in response to HCHF diet. Fig 1 shows unsupervised hierarchical clustering of gene expressionprofiles in low and high LDL-C baboons.

**Table 2:** Low LDL-C baboons: Top 35 genes that exhibit greatest change in response to HCHF diet.

Up-regulated			Down-regulated		
Gene symbol	Corrected p-value	Fold-Change	Gene symbol	Corrected p-value	Fold-Change
**U4**	0.014	4.2	LOC101026757	0.040	-35.7
**IGKV1OR2-108**	0.003	3.0	LOC101017602	0.079	-33.1
**ENKUR**	0.011	2.9	ALB	0.041	-29.7
**TMEM40**	0.006	2.8	CPS1	0.034	-28.7
**GEMIN7**	0.001	2.7	PLG	0.069	-27.0
**NRBF2**	0.037	2.7	HRG	0.017	-26.6
**PPIL3**	0.003	2.7	FGG	0.071	-25.5
**RGS16**	0.056	2.7	LOC101022486	0.045	-22.4
**IGKV4-1**	0.018	2.6	ITIH2	0.074	-22.2
**MRPL47**	0.004	2.6	TTR	0.035	-20.2
**LOC101019904**	0.005	2.5	LOC101023648	0.022	-18.6
**MEX3B**	0.052	2.5	ITIH1	0.020	-18.2
**BORCS5**	0.004	2.4	LOC100998199	0.012	-16.3
**LOC101009271**	0.046	2.4	LOC101000951	0.046	-16.2
**TMEM177**	0.001	2.4	TAT	0.030	-16.0
**CLEC9A**	0.002	2.4	C9	0.076	-15.7
**NRARP**	0.025	2.4	HMGCS2	0.074	-15.2
**NINJ1**	0.015	2.4	AGT	0.053	-14.2
**MRPL50**	0.005	2.3	ADH4	0.047	-13.3
**CCL22**	0.001	2.3	SLCO1B1	0.048	-11.9
**SGPP1**	0.017	2.3	C2	0.021	-11.7
**GPR171**	0.065	2.3	C5	0.015	-11.5
**ND3**	0.022	2.3	PON1	0.010	-11.0
**GRPEL2**	0.009	2.3	LOC101017967	0.069	-10.7
**CCDC102A**	0.006	2.3	C6	0.020	-10.3
**SLAMF9**	0.008	2.3	APOC3	0.062	-10.3
**TROVE2**	0.024	2.3	ARG1	0.008	-9.8
**ST6GALNAC3**	0.048	2.3	LOC100998476	0.071	-9.4
**PPA2**	0.007	2.3	SLC2A2	0.066	-9.4
**BCL10**	0.003	2.3	SERPING1	0.064	-9.1
**COPS4**	0.009	2.3	LOC101001292	0.013	-8.5
**LAMTOR1**	0.007	2.2	AFM	0.062	-8.4
**HOXB7**	0.002	2.2	OTC	0.008	-8.3
**MBD5**	0.012	2.2	SERPINF2	0.060	-8.2
**TCEAL1**	0.005	2.2	LOC101002090	0.031	-7.8

**Table 3:** High LDL-C baboons: Top 35 genes that exhibit greatest change in response to HCHF diet.

Up-regulated			Down-regulated		
Gene symbol	Corrected p-value	Fold-Change	Gene symbol	Corrected p-value	Fold-Change
**ALDOB**	0.026	36.0	HRH2	0.011	-1.8
**LRP2**	0.046	20.9	BCL9L	0.039	-1.8
**SLC34A1**	0.037	18.7	MAML3	0.033	-1.8
**SLC22A2**	0.031	14.1	IKZF1	0.038	-1.8
**IGFBP5**	0.034	11.5	GAS7	0.037	-1.8
**EPHX2**	0.022	9.9	FAM129C	0.040	-1.9
**AGXT2**	0.032	9.6	NIPAL3	0.011	-1.9
**SLC22A6**	0.010	9.4	IQSEC1	0.026	-1.9
**IGFBP7**	0.037	9.1	EXTL3	0.037	-1.9
**HSD11B2**	0.038	7.7	FAM102B	0.039	-1.9
**TSPAN1**	0.027	6.8	CUL9	0.024	-1.9
**CALB1**	0.035	5.9	MGAT3	0.042	-1.9
**SLC6A18**	0.032	5.6	XKRX	0.004	-1.9
**LGALS2**	0.036	5.3	CR2	0.003	-1.9
**SLC47A2**	0.047	5.0	NUDCD2	0.046	-1.9
**SLC3A1**	0.026	4.7	LY96	0.023	-1.9
**OSGIN1**	0.048	4.5	KIAA1549L	0.001	-2.0
**MIOX**	0.034	4.5	TCHP	0.003	-2.0
**SDC4**	0.040	4.4	TXNL4B	0.028	-2.0
**CGNL1**	0.008	4.3	PLEKHA2	0.038	-2.0
**COL4A2**	0.045	4.3	ST6GAL1	0.044	-2.0
**SLC12A3**	0.024	4.3	TRIM25	0.036	-2.1
**SPARC**	0.009	3.4	TMED8	0.021	-2.1
**KCNJ15**	0.040	3.4	BACH2	0.008	-2.1
**GPT**	0.027	3.3	CSPG4	0.011	-2.1
**HNF1B**	0.042	3.3	AFF3	0.027	-2.1
**NOX4**	0.040	3.3	AGBL2	0.003	-2.2
**DPYS**	0.045	3.2	FCRL5	0.028	-2.2
**ESRRG**	0.036	3.1	ROBO2	0.030	-2.2
**CES2**	0.005	3.0	TET3	0.010	-2.2
**USH1C**	0.009	2.9	KIF26A	0.002	-2.3
**VEGFA**	0.005	2.8	ELMSAN1	0.001	-2.3
**CTSL**	0.011	2.7	LIPN	0.009	-2.3
**SERPINH1**	0.044	2.6	SWAP70	0.001	-2.5
**DMGDH**	0.039	2.6	NFATC2	0.001	-2.8

**Fig 2:**
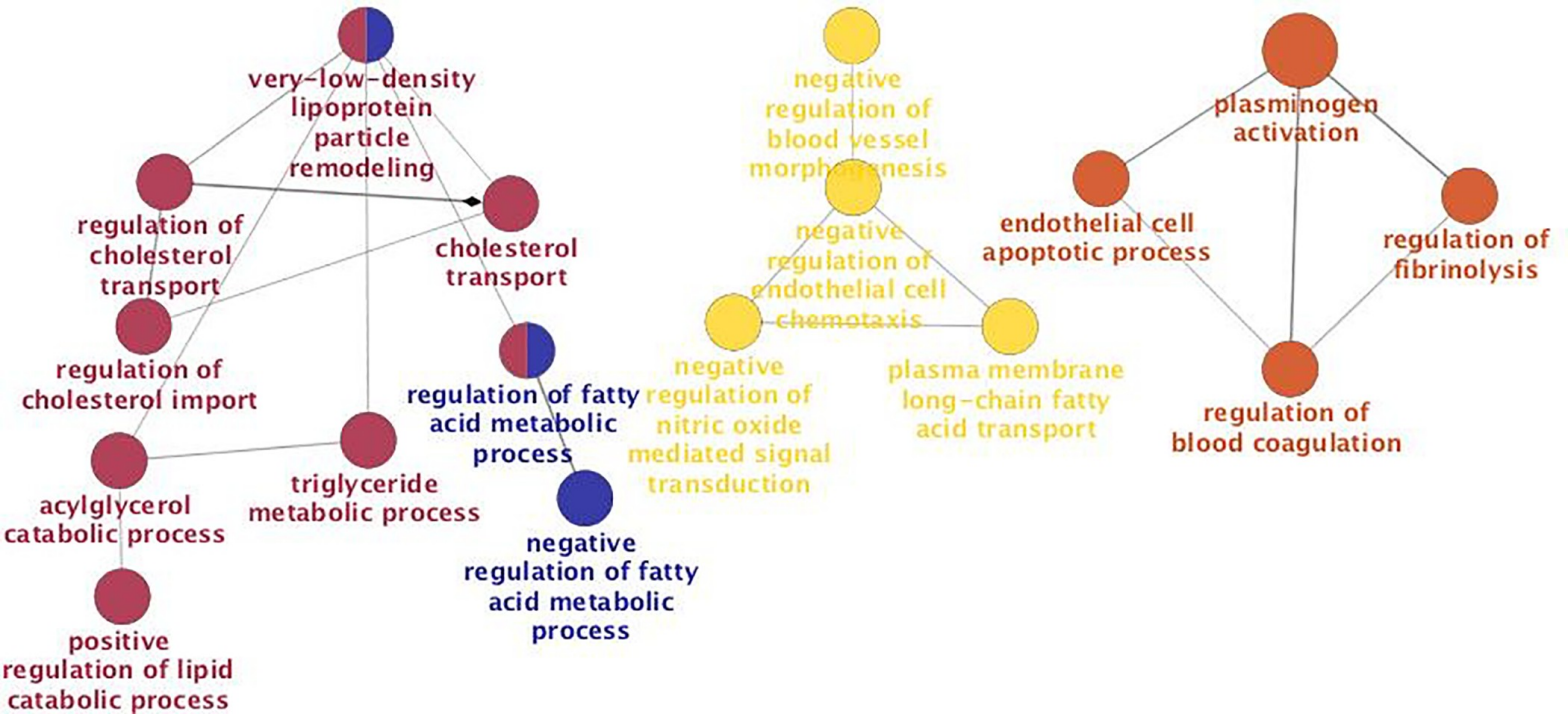
GO/pathway terms networks enriched for differentially expressed genes in low LDL-C baboons, respectively. Terms are represented as nodes linked based on their kappa score 0.4, where only the label of the most significant term per group is shown. The node size represents the term enrichment significance. The node color denotes different GO terms, and color gradient shows the gene proportion of each cluster associated with the term.

**Fig 3:**
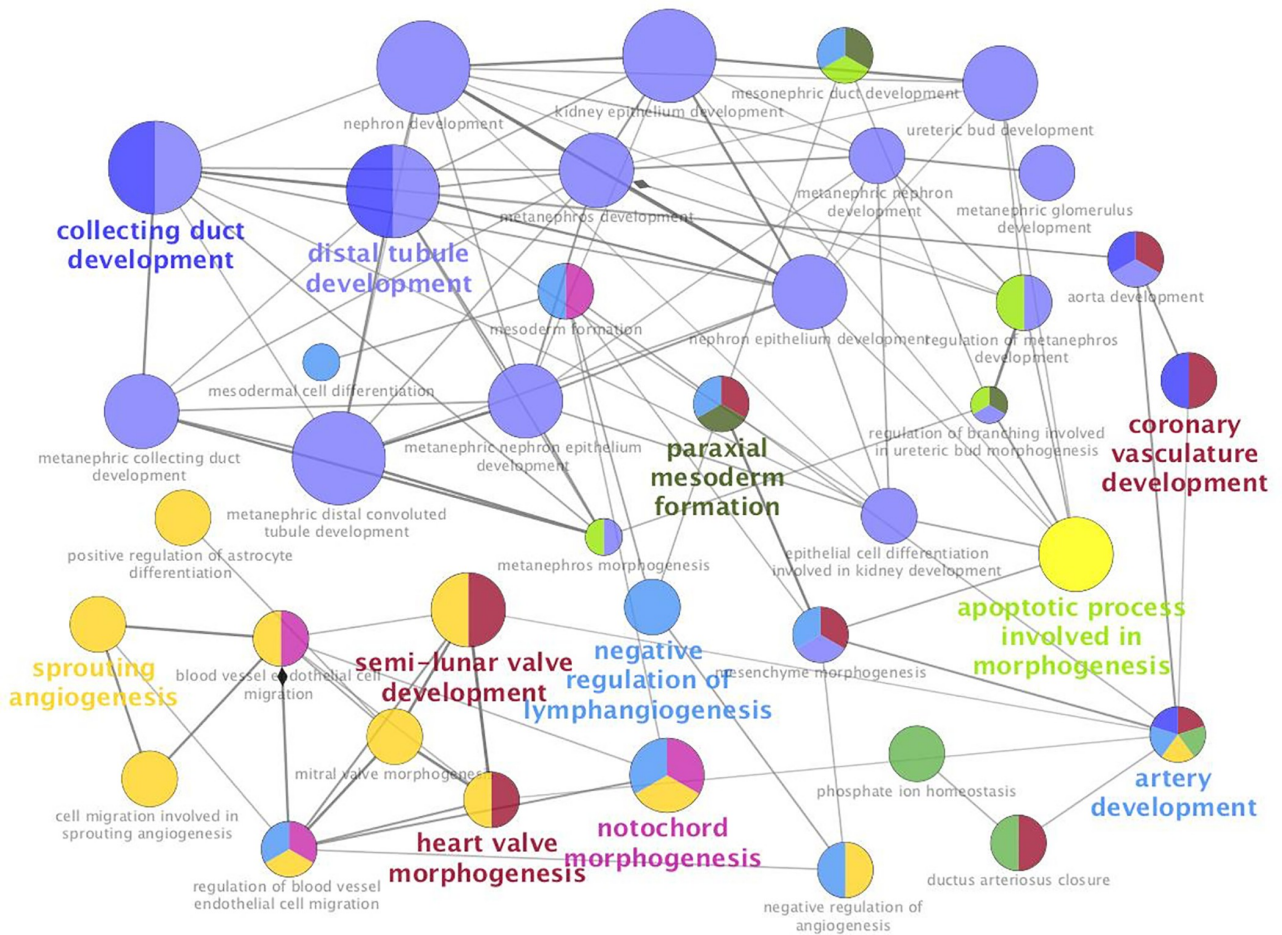
GO/pathway terms networks enriched for differentially expressed genes in high LDL-C baboons, respectively. Terms are represented as nodes linked based on their kappa score 0.4, where only the label of the most significant term per group is shown. The node size represents the term enrichment significance. The node color denotes different GO terms, and color gradient shows the gene proportion of each cluster associated with the term.

Moreover, we used ClueGO (http://apps.cytoscape.org/apps/cluego), an application for Cytoscape, to identify networks of both gene ontology (GO) and pathways influenced by genes differentially expressedin low and high LDL-C baboons. Fig 2 shows functionally grouped networks of GO terms and pathways associated with expressed genes in low LDL-C baboons, including VLDL particle remodeling, regulation of cholesterol transport, endothelial cell apoptotic process, and negative regulation of blood vessel morphogenesis. For high LDL-C baboons, GO terms and pathways included coronary vasculature development, heart valve morphogenesis, kidney epithelium development, distal tubule and collecting duct development (Fig 3).

### Genes expressed in both low and high LDL-C baboons

**Fig 4:**
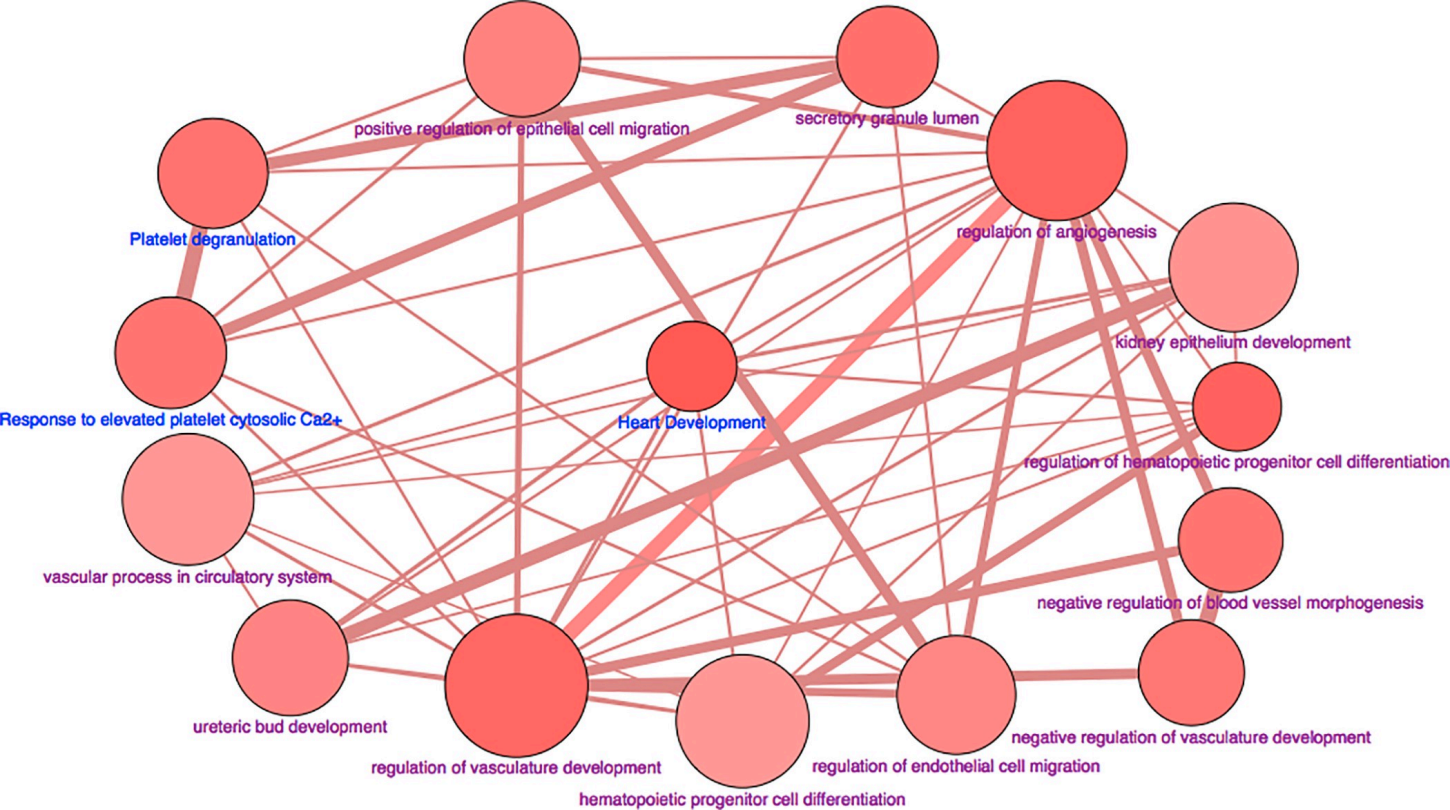
Networks of GO/pathway terms for differentially expressed genes common to low and high LDL-C baboons. Terms are represented as nodes linked based on their kappa score 0.4, where only the label of the most significant term per group is shown. The node size represents the term enrichment significance. The color gradient shows the proportion of the number of genes for each cluster associated with the term.

Eighteen differentially expressed genes were common in both low and high LDL-C baboons (Table 4). After p-value correction, 14 genes remained significantly differentially expressed in both groups. Among the 14 genes, 3 genes (*VEGFA*, *SPARC and DMGDH*) were inversely expressed between the two groups. MAML3, PKD1L3 and LIN9 were inversely expressed between the phenotypes but their differential expression was nominal in low LDL-C baboons. Fig 4 shows a network of GO and pathways associated with the differentially expressed genes. The top GO terms that were statistically significant included regulation of angiogenesis, regulation vascular development, regulation of hematopoietic progenitor cell differentiation, and kidney endothelial angiogenesis.

**Table 4:** Common PBMC genes differentially expressed in low and high LDL-C baboons.

Gene ID	Low LDL-C baboons			High LDL-C baboons		
	Fold-	P-value	Corrected	Fold-	P-value	Corrected
			
	change		P-value	change		P-value
***VEGFA***	-2.59	0.005	0.012	2.77	0.005	0.005
***DMGDH***	-2.63	0.024	0.043	2.64	0.037	0.039
**SPARC**	-2.42	0.027	0.047	3.43	0.009	0.009
***MAML3***	1.71	0.04	0.072	-1.83	0.031	0.033
**PKD1L3**	1.19	0.019	0.063	-1.19	0.035	0.036
**LIN9**	1.54	0.043	0.075	-1.54	0.029	0.031
**GFRA2**	1.33	0.002	0.004	1.21	0.031	0.033
**PRMT8**	1.33	0.037	0.064	1.36	0.044	0.047
**SLAMF9**	2.27	0.004	0.008	1.69	0.028	0.03
**SNORD124**	-1.23	0.019	0.02	-1.23	0.037	0.04
**TMEM203**	1.67	0.021	0.02	1.62	0.031	0.033
**UBN2**	-1.71	0.021	0.02	-1.78	0.022	0.022
**7SK**	1.34	0.002	0.004	1.8	0.041	0.043
**AK5**	1.94	0.013	0.01	1.72	0.025	0.03
**CLEC4E**	1.61	0.044	0.041	1.79	0.006	0.006
**DDN**	1.62	0.005	0.012	1.51	0.044	0.047
**DMRT2**	1.51	0.012	0.01	1.48	0.021	0.021
**FOXC1**	1.59	0.034	0.031	1.54	0.025	0.03

### Annotation of small RNAs and identification of novel miRNAs

**Fig 5:**
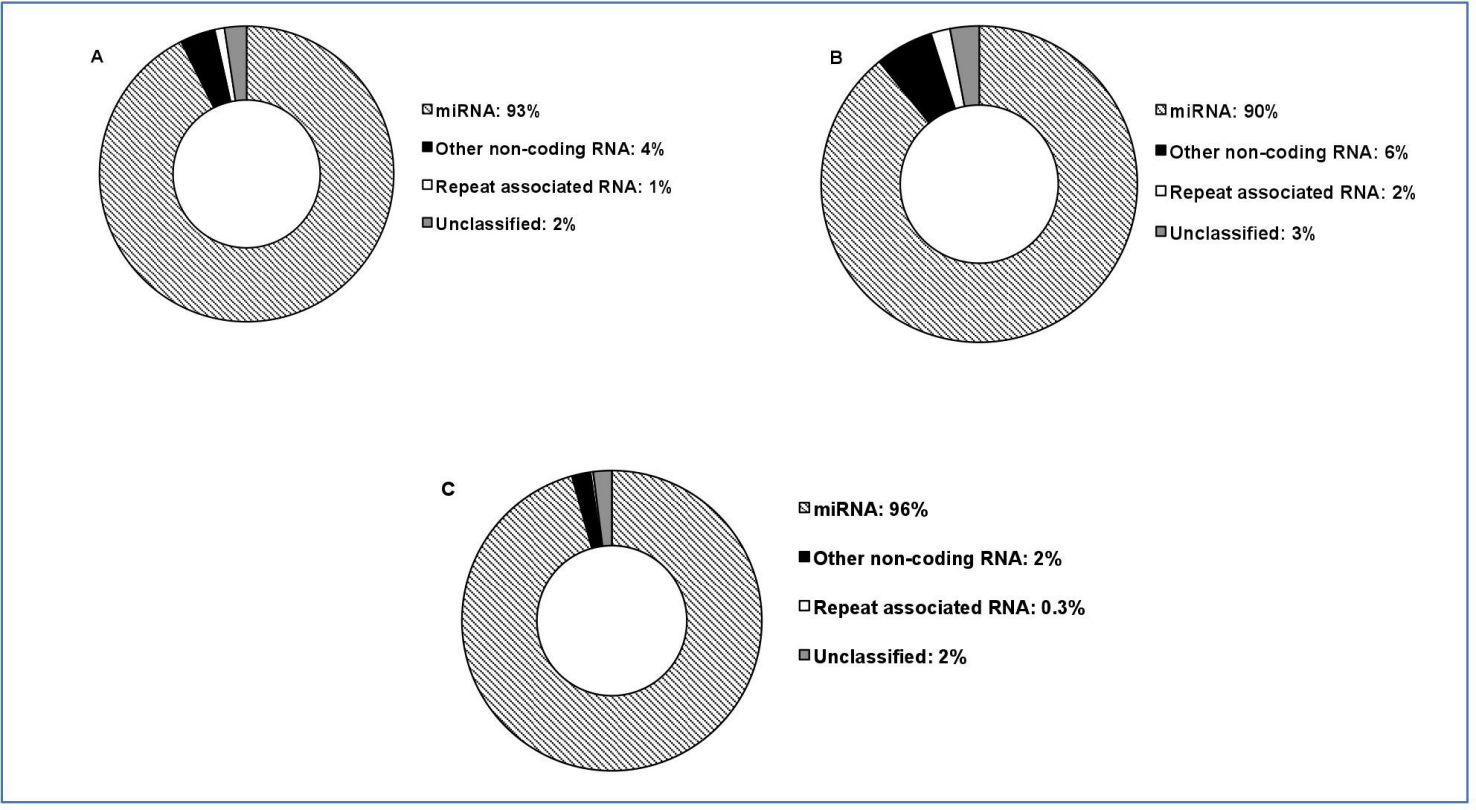
**Proportion of sncRNAs in a) both low and high LDL-C, b) high LDL-C only and c) low LDL-C only PBMC samples.** Segments in the donut diagram represent the proportions of different categories of small RNAs. Notably, “other non-coding RNAs” include snoRNAs, tRNAs, siRNAs,and snRNAs.

The proportion of expressed sncRNAs for each type of annotated sncRNA is shown in Fig 5. Fig 5A shows combined data for low and high LDL-C baboons. miRNAs were the most abundantly expresse of the annotated sncRNAs (93%). Other sncRNAs, such as small interfering RNAs (siRNAs), small nucleolar RNAs (snoRNAs), small nuclear RNAs (snRNAs), and transfer RNAs (tRNAs), comprised 4% of the total expressed sncRNAs. Repeat-associated RNAs accounted for only 1% of the sncRNAs.

Unique sncRNAs that did not map (“unclassified”) to any sncRNA databases or to the human genome comprised approximately 2% of the sncRNAs expressed in all baboon PBMC libraries. Fig 5B and 5 show the data, respectively, for low LDL-C baboons and high LDL-C baboons. A higher proportion of miRNAs were expressed in low LDL-C baboons (96%) compared with high LDL-C baboons (90%).However, the difference was not statistically significant. Other non-coding RNAs accounted for only 2% of the total expressed sncRNAs in low LDL-C baboons and 6% for high LDL-C baboons (Fig 4B and 4C).

We identified 541 distinct miRNAs in baboon PBMCs: 494 were identical to human miRNAs, herein referred to as known miRNAs, and 47 were novel baboon miRNAs (Table 5). A greater number of known and novel miRNAs were expressed in low LDL-C than high LDL-C baboons. All known and novel miRNAs are shown in S2 and S3 Tables.

**Table 5:** PBMC miRNAs expressed in baboons.

Phenotype	Human orthologous miRNAs	Novel baboon miRNAs
Low LDL-C only	35	17
High LDL-C only	23	15
Common to low and high LDL-C	436	15
Total	494	47

### Differential expression of PBMC miRNAs in response to a HCHF diet

**Fig 6:**
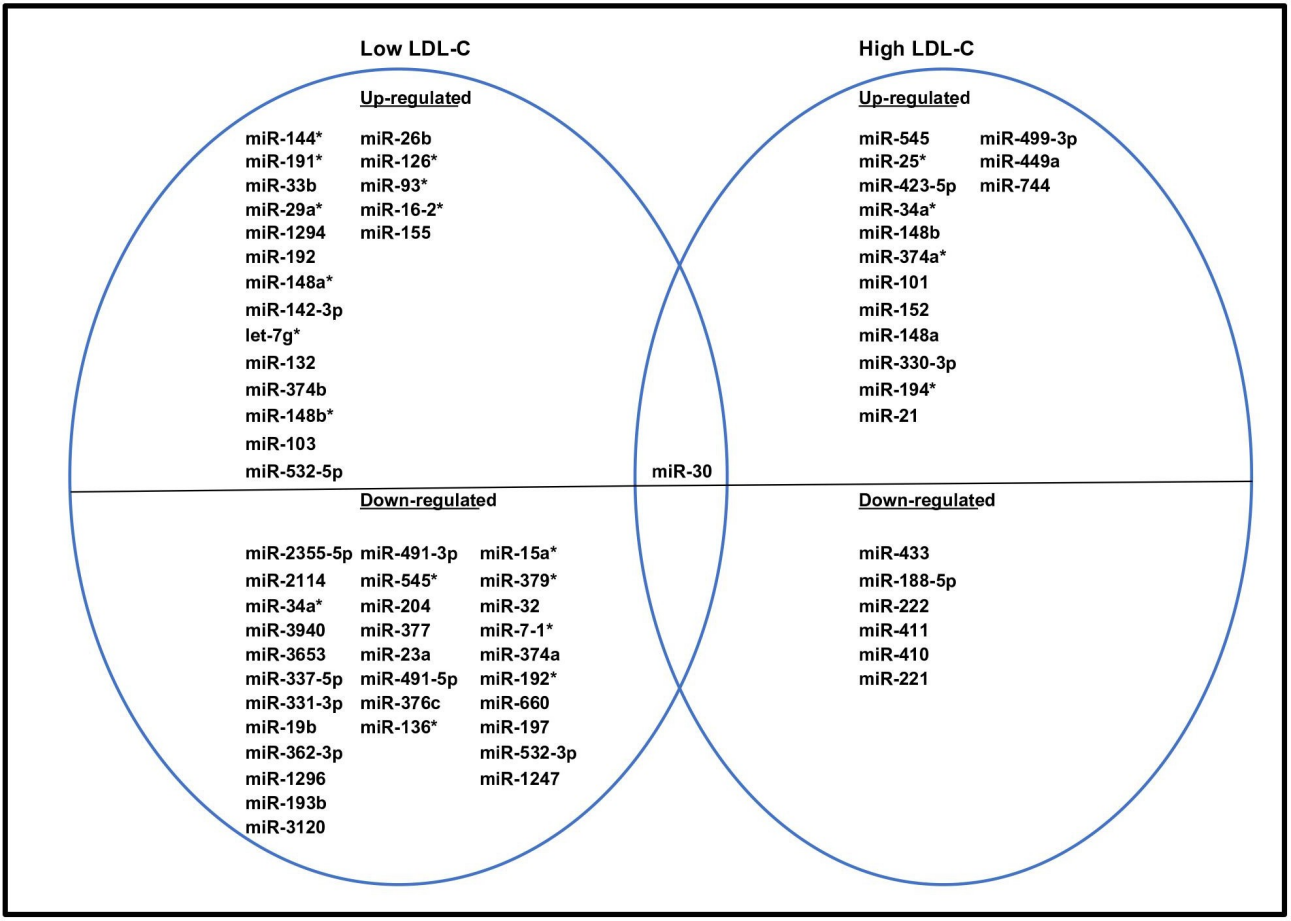
A Venn diagram showing PBMC miRNAs differentially expressed in response to HCHF diet in low and high LDL-C baboons.

MiRNAs that were differentially expressed in response to a HCHF diet in low and high LDL-C baboons are shown in Fig 6. Fifty miRNAs were differentially expressed in low LDL-C baboons (21 up- and 29 down-regulated). For high LDL-C baboons, 20 miRNAs were differentially expressed in response to LDL- C level change (11 up- and 9 down-regulated). The miR-30 family was down-regulated in both low and high LDL-C baboons. Notable miRNAs up-regulated in low LDL-C PBMCs but not significantly differentially expressed in high LDL-C baboons included miR-33b, 29b, 16, 155 and 26b. The miR- 221/222 family was down-regulated in high LDL-C baboons but not significantly differentially expressed in low LDL-C samples. Table 6 shows the miRNA nucleotide sequences, p values and fold changes of these miRNAs.

**Table 6:** Differentially expressed PBMC miRNAs in response to HCHF diet in low and high LDL-C baboons.

miRNA-ID	p-value	Fold-change	mature miRNA sequence
High LDL-C baboons			
miR-545-3p	0.04	322.54	UCAGCAAACAUUUAUUGUGUGC
miR-25-5p	0.01	7.28	AGGCGGAGACUUGGGCAAUUG
miR-423-5p	0.00	7.06	UGAGGGGCAGAGAGCGAGACUUU
miR-34a-3p	0.05	4.48	CAAUCAGCAAGUAUACUGCCCU
miR-148b-3p	0.00	4.20	UCAGUGCAUCACAGAACUUUGU
miR-374a-3p	0.02	4.19	CUUAUCAGAUUGUAUUGUAAUU
miR-101-3p	0.05	4.01	UACAGUACUGUGAUAACUGAA
miR-152-3p	0.04	3.93	UCAGUGCAUGACAGAACUUGG
miR-148a-3p	0.04	3.75	UCAGUGCACUACAGAACUUUGU
miR-330-3p	0.03	3.59	GCAAAGCACACGGCCUGCAGAGA
miR-194-3p	0.03	3.20	CCAGUGGGGCUGCUGUUAUCUG
miR-21-5p	0.05	2.99	UAGCUUAUCAGACUGAUGUUGA
miR-499-3p	0.03	2.67	AACAUCACAGCAAGUCUGUGCU
miR-449a	0.05	2.53	UGGCAGUGUAUUGUUAGCUGGU
miR-744-5p	0.03	2.29	UGCGGGGCUAGGGCUAACAGCA
miR-433-3p	0.00	-0.73	AUCAUGAUGGGCUCCUCGGUGU
miR-188-5p	0.04	-1.53	CAUCCCUUGCAUGGUGGAGGG
miR-222-3p	0.05	-1.61	AGCUACAUCUGGCUACUGGGU
miR-411-5p	0.01	-1.64	UAGUAGACCGUAUAGCGUACG
miR-410-3p	0.04	-1.65	AAUAUAACACAGAUGGCCUGU
miR-221-3p	0.03	-1.68	AGCUACAUUGUCUGCUGGGUUUC
Low LDL-C baboons			
miR-144-5p	0.01	16.20	GGAUAUCAUCAUAUACUGUAAG
miR-191-3p	0.04	7.21	GCUGCGCUUGGAUUUCGUCCCC
miR-33b-5p	0.05	6.56	GUGCAUUGCUGUUGCAUUGC
miR-29a-5p	0.04	5.68	ACUGAUUUCUUUUGGUGUUCAG
miR-1294	0.00	4.58	UGUGAGGUUGGCAUUGUUGUCU
miR-192-5p	0.04	4.39	CUGACCUAUGAAUUGACAGCC
miR-148a-5p	0.01	4.28	AAAGUUCUGAGACACUCCGACU
miR-142-3p	0.05	3.53	UGUAGUGUUUCCUACUUUAUGGA
let-7g-3p	0.04	3.27	CUGUACAGGCCACUGCCUUGC
miR-132-3p	0.04	3.13	UAACAGUCUACAGCCAUGGUCG
miR-374b-5p	0.01	3.02	AUAUAAUACAACCUGCUAAGUG
miR-148b-5p	0.01	3.01	AAGUUCUGUUAUACACUCAGGC
miR-103a-3p	0.01	2.92	AGCAGCAUUGUACAGGGCUAUGA
miR-532-5p	0.01	2.85	CAUGCCUUGAGUGUAGGACCGU
miR-26b-5p	0.02	2.84	UUCAAGUAAUUCAGGAUAGGU
miR-126a-5p	0.04	2.69	CAUUAUUACUUUUGGUACGCG
miR-93-3p	0.03	2.43	ACUGCUGAGCUAGCACUUCCCG
miR-16-2-3p	0.01	2.42	CCAAUAUUACUGUGCUGCUUUA
miR-155-5p	0.04	2.31	UUAAUGCUAAUCGUGAUAGGGGU
miR-2355-5p	0.00	-1.08	AUCCCCAGAUACAAUGGACAA
miR-2114-5p	0.01	-1.10	UAGUCCCUUCCUUGAAGCGGUC
miR-34a-3p	0.04	-1.12	CAAUCAGCAAGUAUACUGCCCU
miR-3940-3p	0.02	-1.16	CAGCCCGGAUCCCAGCCCACUU
miR-3653-3p	0.01	-1.19	CUAAGAAGUUGACUGAAG
miR-337-5p	0.04	-1.22	GAACGGCUUCAUACAGGAGUU
miR-331-3p	0.05	-1.22	GCCCCUGGGCCUAUCCUAGAA
miR-19b-3p	0.01	-1.25	UGUGCAAAUCCAUGCAAAACUGA
miR-362-3p	0.02	-1.25	AACACACCUAUUCAAGGAUUCA
miR-1296-5p	0.04	-1.26	UUAGGGCCCUGGCUCCAUCUCC
miR-193b-3p	0.04	-1.27	AACUGGCCCUCAAAGUCCCGCU
miR-3120-5p	0.03	-1.33	CACAGCAAGUGUAGACAGGCA
miR-491-3p	0.00	-1.37	CUUAUGCAAGAUUCCCUUCUAC
miR-545-5p	0.05	-1.37	UCAGUAAAUGUUUAUUAGAUGA
miR-204-5p	0.01	-1.38	UUCCCUUUGUCAUCCUAUGCCU
miR-377-3p	0.02	-1.41	AUCACACAAAGGCAACUUUUGU
miR-23a-3p	0.04	-1.42	AUCACAUUGCCAGGGAUUUCC
miR-491-5p	0.03	-1.42	AGUGGGGAACCCUUCCAUGAGG
miR-376c-3p	0.04	-1.43	AACAUAGAGGAAAUUCCACGU
miR-136-3p	0.03	-1.44	CAUCAUCGUCUCAAAUGAGUCU
miR-15a-5p	0.02	-1.47	UAGCAGCACAUAAUGGUUUGUG
miR-379-3p	0.04	-1.49	UAUGUAACAUGGUCCACUAACU
miR-32-5p	0.05	-1.49	UAUUGCACAUUACUAAGUUGCA
miR-7-1-3p	0.01	-1.55	CAACAAAUCACAGUCUGCCAUA
miR-374a-5p	0.02	-1.57	UUAUAAUACAACCUGAUAAGUG
miR-192-3p	0.05	-1.57	CUGCCAAUUCCAUAGGUCACAG
miR-660-5p	0.01	-1.63	UACCCAUUGCAUAUCGGAGUUG
miR-197-3p	0.00	-1.63	UUCACCACCUUCUCCACCCAGC
miR-532-3p	0.01	-1.65	CCUCCCACACCCAAGGCUUGCA
miR-1247-5p	0.04	-1.70	ACCCGUCCCGUUCGUCCCCGGA
Both low and high LDL-C baboons			
miR-30-5p	0.04	3.52	UGUAAACAUCCUCGACUGGAAG

We identified star miRNAs (complementary sequence to mature miRNA) that were differentially expressed and exhibited discordant expression in low and high LDL-C baboons. MiR-34a* and 374a* (miR-34a-3p and 374a-3p) were up-regulated in low LDL-C baboons and down-regulated in high LDL-C baboons. Interestingly, miR-148a and miR-148b were up-regulated in low LDL-C baboons while complementary sequences, miR-148a* (miR-148a-5p) and miR-148b* (miR-148b-5p), were up-regulated in high LDL-C baboons.

### Targets of differentially expressed miRNAs

We used IPA to identify miRNA gene-targets that are differentially expressed and inversely correlated with differentially expressed miRNAs. Table 7 shows that the total number of targeted genes for low LDL- C baboons was significantly greater than for high LDL-C baboons, z-score = 24.6, p<0.001. These miRNA targets included genes involved in lipid exchange and transport, angiogenesis and glucose homeostasis such as APOL1, *VEGFA*, IGFBP5, and MAMAL3. S4 Table shows all genes that were differentially expressed and inversely correlated with differentially expressed miRNAs in low and high LDL-C baboons.

**Table 7:** Number of miRNA gene-targets inversely correlated with miRNA expression in low and highLDL-C baboons.

Phenotypes	Up-regulated	Down-regulated	Total
Low LDL-C	348	69	417
High LDL-C	9	44	53

### Expression of miRNAs and miRNA-targets is coordinated in response to LDL-C levels

**Fig 7:**
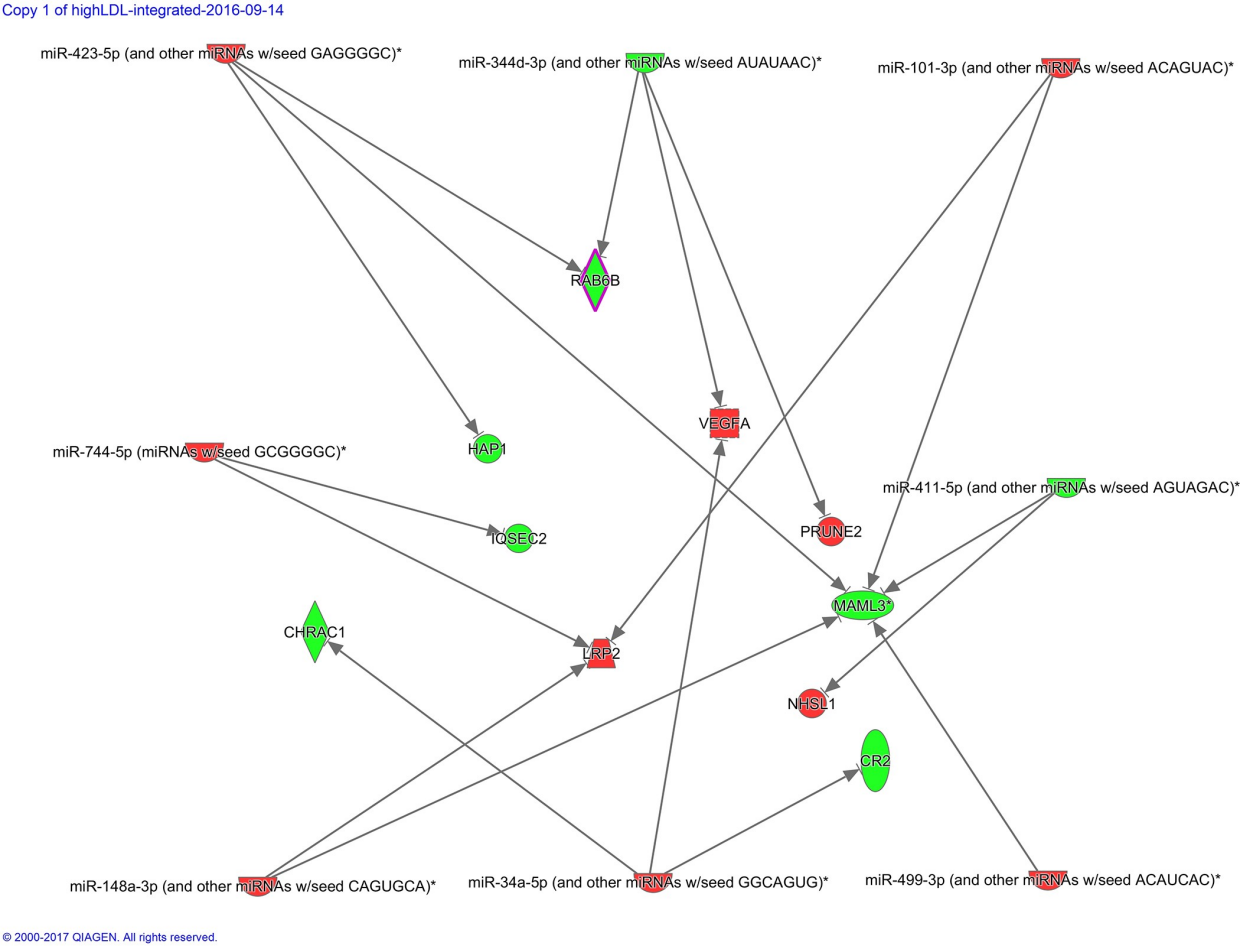
Networks of PBMC miRNAs and miRNA targets that are inversely expressed in low baboons, respectively. Genes and miRNAs are represented as nodes. Red nodes indicate up-regulated genes, and green nodes denote down-regulated miRNAs. The intensity of the color indicates the degree of differential expression. Numerals below each colored node represent expression fold-change and *P*-values. The molecular relationship between nodes is represented as a line (edge); arrows indicate the direction of interaction.

**Fig 8:**
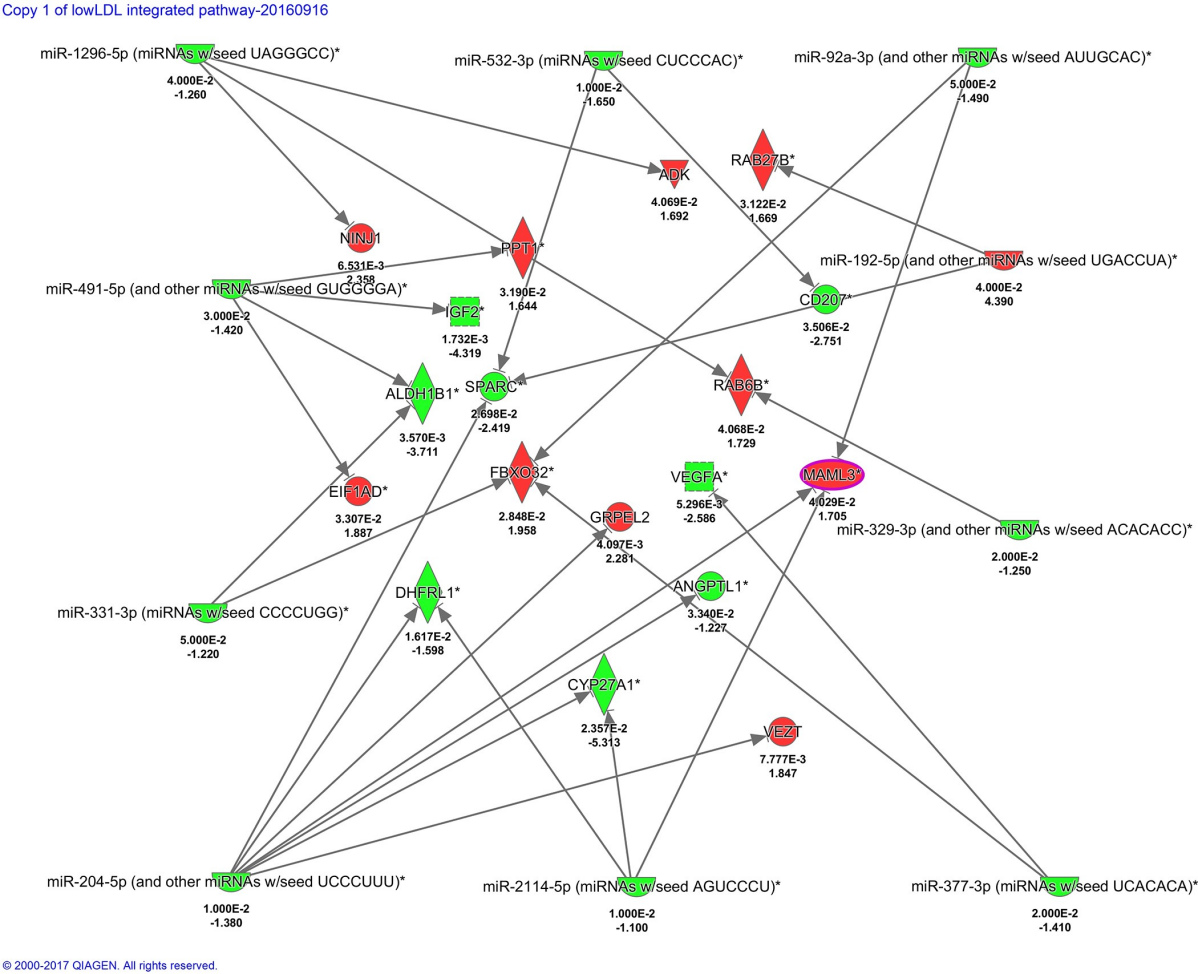
Networks of PBMC miRNAs and miRNA targets that are inversely expressed in high LDL-C baboons, respectively. Genes and miRNAs are represented as nodes. Rednodes indicate up-regulated genes, and green nodes denote down-regulated miRNAs. The intensity of the color indicates the degree of differential expression. Numerals below each colored node represent expression fold-change and *P*-values. The molecular relationship between nodes is represented as a line (edge); arrows indicate the direction of interaction.

We integrated differentially expressed genes and differentially expressed miRNAs in response to HCHF diet in low and high LDL-C baboons. Notably, the genes and miRNAs were identified from the same samples. Figs 7 and 8 show networks of miRNAs and miRNA targets that were inversely expressed in low and in high LDL-C baboons, respectively. We found out that *VEGFA*, *MAML3*, and LRP2 are major hubs that are differentially expressed between low and high LDL-C baboons and inversely expressed in respect to miRNAs that are differentially expressed in low and high LDL–C baboons.

## Discussion

Plasma high LDL-C concentration is a major risk factor for atherosclerosis, the leading cause of CVD. LDL-C contributes greatly to the development and progression of atherosclerosis by inducing the expression of EC adhesion molecules and ameliorating endothelial cell apoptosis. Various studies have shown that LDL-C particles, particularly when oxidized, induce EC apoptosis by altering the expression of miRNAs or miRNA target genes *in vitro* [11, 12]. In addition, PBMCs, particularly monocytes play a key role during initiation of atherogenesis, where they infiltrate vascular intima and differentiate to macrophages, triggering a cascade of inflammatory signals. Further, recent studies have demonstrated that monocyte-derived exosomes transport miRNAs that activate adhesion molecules of EC *in vitro* [22, 23]. Previously, we showed that LDL-C concentrations influence the expression of hepatic miRNAs in response to a HCHF diet [13]. In the current study, we aimed to assess differential expression of PBMC miRNAs and miRNA gene-targets in baboons differing in LDL-C response to a HFHC diet, together with identification of coordinately expressed miRNAs and miRNA gene-targets using unbiased methods. To address these aims we combined high throughput RNA sequencing together with bioinformatics, including TargetScan software embedded in IPA, EM algorithm in Partek package, GO and KEGG pathway analysis. To our knowledge this is the first study to interrogate the expression of miRNAs and miRNA targets in PBMCs in response to a HCHF diet challenge in nonhuman primates discordant forLDL-C response to a dietary challenge.

We observed that 626 genes were differentially expressed in response to a HCHF diet challenge in low LDL baboons and 272 genes in high LDL baboons multiple testing correction. The top 35 gens with highest fold change in low LDL-C baboons includes *CPS*, which catalyzes synthesis of carbamoyl phosphate and mutation in the gene leads to susceptibility to persistent hypertension, and *PLG*, the precursor for plasmin and angiotensin. Mutation in *PLG* leads to thrombophilia. The top 35 genes in high LDL-C baboons includes *ALDOB*, which is a glycolytic enzyme, and a lipoprotein receptor (*LRP2*).

Eighteen genes were differentially expressed in response to HCHF diet in both low LDL-C baboons and high LDL-C baboons. Among the 18 genes, 3 (*VEGFA*, *DMGDH and SPARC*) were inversely expressed between the baboon phenotypes. These PBMC candidate genes whose expression is influenced by dyslipidemia in baboons are potential therapeutic targets. Future studies will validate their expressionand decipher the molecular mechanisms associated with dyslipidemia.

For miRNAs, 70 were differentially expressed; 50 in low LDL-C and 20 in high LDL-C baboons. Notable miRNAs include miR545-3, which exhibited 322-fold change in high LDL-C baboons, miR-221\222, miR- 34a, and 148a/b. Integration of differentially expressed miRNAs, and, inversely, differentially expressed genes targeted by these miRNAs, revealed that the major hubs are regulated by multiple miRNAs. Forexample, in high LDL-C baboons, *VEGFA* is targeted by miR-34a-5p and miR-344d-3p, and *MAML3* is putatively targeted by miR-499, miR-148a-3p, miR-423-5p, miR-101-3p and miR-411-5p. These observations are consistent with previous knowledge that miRNAs coordinately regulate major gene hubs in a complex network by binding at multiple sites to fine tune their expression.

Importantly, we observed differing integrated GO terms and pathways in low and high LDL-C baboon groups. For low LDL-C baboons, the major G0 terms and pathways associated with expressed genes in response to HCHF diet are linked to lipid metabolism. In contrast, GO terms and pathways associated with vascular development, apoptosis and kidney development are enriched for genes expressed in high LDL-C baboons. Genes expressed in both groups are associated with angiogenesis and vascular development. The high plasma cholesterol in high LDL-C baboons is associated with attenuated lipid metabolism pathways and activated epithelial development pathways, possibly in response to endothelium injury modulated by LDL-C.

### Candidate genes and signaling pathways

#### *VEGFA* and *VEGF* signaling pathway

*VEGFA* is a major ligand for the VEGFR1 receptor that is involved in VEGF signaling pathway, implicated in many pathophysiological conditions, including CVD. In our study, the *VEGFA* was down-regulated inlow LDL-C baboons, and up-regulated in high LDL-C baboons. Our findings indicate that the high LDL-C plasma concentrations correlated with increased expression of *VEGFA* in PBMCs.

LDL-C plays a major role initiating atherosclerosis, and one possible mechanism involves up-regulation of *VEGFA*. It’s plausible that PBMC-secreted *VEGFA* may interact with monocyte *VEGFA* receptor to induce atherogenesis-related inflammatory cytokines. In mice, cholesterol loaded in macrophagesinfluenced the up-regulation of *VEGFA* [24]. In humans, *VEGFA* was up-regulated in neointimal lesions compared to control [25]. Future studies will investigate the mechanistic role of *VEGFA* expressed in PBMCs in response to HCHF diet, in relation to atherogenesis, and whether *VEGFA* is a potential biomarker for dyslipidemia.

#### *DMGDH* and choline oxidative pathway

*DMGDH* encodes an enzyme that catalyzes the demethylation of dimethylglycine (DMG), leading to the subsequent formation of sarcosine and glycine in a canonical choline oxidative pathway in the mitochondria [26]. In our study, *DMGDH* was up-regulated in high LDL-C baboons while it was down- regulated in low LDL-C baboons in response to HCHF diet. Our findings suggest that the concentrationof plasma cholesterol influences the expression of *DMGDH*, likely in response to accumulated DMG levels.

Aberrant plasma levels of DMG are associated with various pathophysiological conditions. Elevated DMG plasma levels are associated with atherosclerotic CVD [27] and mortality [28], impaired renal function [29] and CRP-related inflammation [27]. Because DMHDH catalyzes metabolism of DMG, it is likely that increased levels of DMG will be accompanied by increased expression of *DMGDH*. Our results will stimulate further studies to investigate whether *DMGDH* expression is correlated with DMG plasmalevels, and their potential role in the development of atherosclerosis.

#### *SPARC* and TGF/B signaling pathway

*SPARC* encodes an extracellular matrix glycoprotein molecule that is involved in transduction of a plethora of cell signaling pathways, including TGF-beta pathway by interacting with TGF/B receptor II [30–32]. Other pathways regulated by *SPARC* include Wnt/beta-catenin and MAPK pathways [33, 34].

We found that *SPARC* expression was up-regulated in high LDL-C baboons and down-regulated in low LDL-C baboons. Our results corroborate with a previous study which indicated that ox-LDL-C influenced *SPARC* expression [35]. The mechanisms that link high LDL-C and *SPARC* expression are unclear, but it is likely high LDL-C may stimulate expression of *SPARC* in PBMCs. The subsequent secretion of SPARC proteins into circulation may trigger inflammation, vascular endothelial cell apoptosis, and vascular smooth cell proliferation to initiate atherosclerosis.

In other studies, elevated plasma concentrations of SPARC are associated with diabetes mellitus, inflammation and dyslipidemia [36] and obesity [37]—all risk factors for cardiovascular disease. These results correlate with findings that *SPARC* plasma concentrations are elevated in patients with coronary artery disease [38]. In *ex-vivo*, *SPARC* expression was up-regulated in both vascular endothelial cells and smooth muscle cells in atherosclerotic lesions in rhesus macaques (*Macaca mulatta*) [39]. Together,these findings suggest that *SPARC* plays a fundamental role in dyslipidemia and in the development of atherosclerosis.

### Differentially expressed candidate miRNAs and their role in dyslipidemia

#### miR-545-3p

miR-545 is encoded in a long noncoding RNA associated with the X-linked FTX gene (lnc-FTX). In our study, miR-545 had the greatest change in response to HCHF diet challenge with a 322-fold change in high LDL-C baboons comparing the HCHF diet to the baseline chow diet. This is the first study showing association of miR-545 expression with LDL-C concentrations. Our miRNA/mRNA prediction analysis indicated that miR-545 gene-targets include LDLRAP1, LDLRAD4, and VLDLR. Elsewhere, in a study comparing patients with CAD and controls, platelet-associated miR-545 was up-regulated in CAD patients compared to controls [40]. Future studies will validate the interaction of miR-545-3p with its gene-targets and molecular mechanisms underlying dyslipidemia in a baboon model of atherosclerosis.

#### miR-34

We found discordant expression of miR-34a* (miR-34a-3p), whereby the miRNA is up-regulated in high LDL-C baboons but down-regulated in low LDL-C baboons. However, very little is known about the role of miR-34a-3p in lipid metabolism. It is plausible that miR-34a-3p plays an important role in lipid metabolism and atherosclerosis, especially because it is evidently involved in both progenitor and EC apoptosis *in vitro* [41, 42]. The pro-apoptosis nature of miR-34a parallels the LDL-C mediated endothelial cell senescence and may explain the up-regulation of miR-34a-3p in high LDL-C baboons. Hepatic miR- 34a was up-regulated in mice fed a high-fat diet, which led to down-regulation of *PPARα* and *SIRT1* [43]. An increasing body of evidence shows that miR-34a, which is a transcriptional target of p53, has tumor suppressive properties mediating apoptosis, cell cycle arrest, and senescence by inhibiting multiple oncogenic pathways [44, 45]. In some cancers, miR-34a is silenced typically by hypermethylation of its regulatory promoter [46]. A miRNA mimic for miR-34 is currently in clinical trial for patients with advanced solid tumors [44]. Further studies will investigate whether the promoter that regulates mir-34a-3p transcription is differentially methylated between low and high LDL-C baboons.

#### miR-221/222

miR-221/222 genes are located in close proximity on the X-chromosome (Xp11.3) and are regulated in a coordinated manner [47]. In our study, we observed that both miR-221 and miR-222 were down-regulated in high LDL-C baboons in response to HCHF diet in concordance with our previous findings for the hepatic microRNAome for the same animals [13].

Recent evidence suggests that both miR-221 and 222 are anti-apoptotic and silenced by high LDL-C concentrations. Both miR-221 and miR-222 were down-regulated in oxidized LDL-C-induced apoptotic EC *in vitro* [12], and exogenous inhibition of miR-221/221 led to enhanced EC apoptosis by up-regulating Est1 and p21 [11]. A contrasting report showed that miR-221/222 plays a pro-apoptotic role in EC in serum-deprived media [48]. Importantly, serum-deprived media may not accurately represent the *in vivo* physiological condition. In comparison, ox-LDL-C-induced EC morphological changes recapitulate similar events during atherosclerosis *in vivo* [49]. This discrepancy may suggest that the functional role of a miRNA depends on environmental context. Our data suggest that high LDL-C may influence the down-egulation of expression of miR-221/222 in PBMCs, most likely to antagonize the miRNA anti-apoptotic functional role, and to promote initialization of atherosclerosis, in concordance with findings in *in-vitro* experiments.

#### miR-148

miR-148 is a promising candidate that plays an important role in regulating lipidemia [50, 51]. We observed a novel discordant expression of mature miR-148a/b-3p and complementary ‘star’ sequences (miR-148a/b-5p) in low and high LDL-C baboons. miR-148a/b-3p were up-regulated in low LDL-C while miR148a/b-5p were up-regulated in high LDL-C baboons. This is the first study to report discordant expression of mature miR-148 and complementary sequences. These findings suggest that miR-148 and its complementary sequences may play different roles in low and high LDL-C concentrations and are potential novel therapeutic targets for dyslipidemia and atherosclerosis.

Two recent studies have identified miR-148a as a negative regulator of LDLR expression and activity [51, 52]. In mice, repression of miR-148a increased clearance of circulating labeled LDL particles and decreased LDL-C levels [51]. In addition, miR-148a is a critical regulator of HDL biogenesis and cholesterol efflux by targeting ABCA1, which plays an important role in removal of excess cholesterol from peripheral cells, including macrophages in atherosclerotic plaques [50]. Thus, miR-148a may play a unique role of decreasing LDL-C and increasing HDL particles.

In conclusion, we have demonstrated molecular genetic differences in response to a HCHF diet; these responses are evident in expression levels of individual genes and miRNAs, as well as in expression of coordinated networks of genes and miRNAs. The miRNAs and miRNA gene-targets differentially expressed in baboon with low LDL response versus high LDL response to a HCHF diet are potential therapeutic targets for lowering LDL-C and alleviating atherosclerosis burden. Although existing therapies are effective in lowering LDL-C concentrations and reducing the progression of atherosclerotic plaques in many people, the risk factors and incidence of CVD are still alarmingly high, and for some patients, the treatments are either not effective or tolerable. The observed genetic differences may explain why current therapies are not effective in some individuals. Thus, there is a need for a continued search for novel therapies. Interestingly, miRNAs are being considered for treatment because of their specificity and reduced side effects [44, 53]. Further studies are necessary to understand the molecular mechanisms by which high LDL-C alters the expression of genes and miRNAs in PBMCs, and whether there is a link between the expression profiles and the initiation of atherosclerosis. Such initiatives will enable delivery of a cassette of miRNA mimics or inhibitors to facilitate fine-tuning of gene networks, rather than delivery of single candidate genes. While miRNA therapeutics raise concerns over potential off-target effects current work on miR-122 has demonstrated that these therapies may prove more effective than, or complement, existing treatment options for CVD. Because nonhuman primates and humans are phylogenetically close, and are similar in their physiology and genetics, the baboon findings will be directly translatable to humans.

## Supporting information

S1 Table Expressed genes in low and high LDL-C baboons v2. (XLSX)

S2 Table All_human orthologous_miRNAs. (XLS)

S3 Table All_baboon_novel_miRNAs. (XLS)

S4 Table Inversely correlated miRNAs-genes in low and high LDL-C baboons. (XLSX)
